# A New Rise of Non-Human Primate Models of Synucleinopathies

**DOI:** 10.3390/biomedicines9030272

**Published:** 2021-03-09

**Authors:** Margaux Teil, Marie-Laure Arotcarena, Benjamin Dehay

**Affiliations:** Université de Bordeaux, CNRS, IMN, UMR 5293, F-33000 Bordeaux, France; margaux.teil@u-bordeaux.fr (M.T.); marie-laure.arotcarena@u-bordeaux.fr (M.-L.A.)

**Keywords:** non-human primates, synucleinopathy, neurodegeneration, α-synuclein, animal model

## Abstract

Synucleinopathies are neurodegenerative diseases characterized by the presence of α-synuclein-positive intracytoplasmic inclusions in the central nervous system. Multiple experimental models have been extensively used to understand better the mechanisms involved in the pathogenesis of synucleinopathy. Non-human primate (NHP) models are of interest in neurodegenerative diseases as they constitute the highest relevant preclinical model in translational research. They also contribute to bringing new insights into synucleinopathy’s pathogenicity and help in the quest and validation of therapeutical strategies. Here, we reviewed the different NHP models that have recapitulated key characteristics of synucleinopathy, and we aimed to highlight the contribution of NHP in mechanistic and translational approaches for synucleinopathies.

## 1. Introduction

Synucleinopathies are neurodegenerative diseases characterized by α-synuclein (α-syn)-positive intracytoplasmic inclusions, which accumulate in the central nervous system (CNS) but are also found in the peripheral and enteric nervous (ENS) systems. The synucleinopathy family includes Parkinson’s disease (PD), Dementia with Lewy Bodies (DLB), and Multiple system atrophy (MSA). Each pathology differs from the other clinically and neuropathologically by the different brain areas most affected and the specific cell type targeted by α-syn accumulation. Parkinson’s disease, the most prevalent synucleinopathy [1], is characterized by a parkinsonian motor syndrome associated with (i) a striatal dopaminergic deficit due to the loss of dopaminergic neurons in the substantia nigra pars compacta (SN) and (ii) the presence of α-syn-positive intracytoplasmic inclusions present in the neurons and widespread in the CNS, called Lewy Bodies (LB) [2,3]. The second most prevalent synucleinopathy, DLB [4], differs from PD clinically with the appearance of early cognitive impairments in parallel with motor symptoms and neuropathologically with amyloid plaques in cortical areas in addition to widespread LB accumulation in the brain [5]. MSA is a rarer synucleinopathy in terms of prevalence [6]. MSA is clinically separated into two phenotypes: MSA-parkinsonian with a parkinsonian syndrome associated with nigrostriatal dopaminergic loss, and MSA-cerebellar with cerebellar syndrome with olivopontocerebellar loss. MSA differs from PD and DLB by the presence of α-syn-positive intracytoplasmic inclusions accumulated in oligodendrocytes named Glial Cytoplasmic Inclusions (GCI) [7].

**Figure 1:**
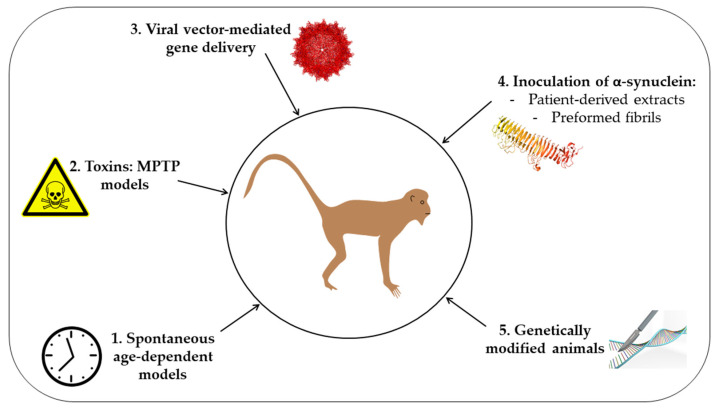
Schematic representation of the different approaches used to generate non-human primate models of synucleinopathy. 1. Spontaneous age-dependent models; 2. Toxins: MPTP models; 3. Viral-mediated gene delivery; 4. Inoculation of α-synuclein using either patient-derived extracts or preformed fibrils; 5. Genetically-modified animal models.

To better understand synucleinopathies’ pathophysiology and propose therapeutic strategies to alter the disease progression, appropriate animal models should be used. An ideal model to study synucleinopathy must recapitulate the different characteristics of the disease, including the course of the disease, the neurodegeneration, and the distribution and accumulation of α-syn-positive intracytoplasmic inclusions. Among experimental models, non-human primates (NHP) to study neurological disorders can present different advantages. Primates present two distinct phylogenetic groups: Old World monkeys and New World monkeys. Both groups are studied in research and present various benefits in terms of laboratory use and human similarities. Phylogenetically, Old World monkeys are the closest species to humans, including macaques and baboon monkeys [8]. Humans are also close to the New World monkeys’ group, including squirrel monkeys and marmosets, as they shared a common phylogenetic ancestor [8]. NHPs are also closest to humans in terms of anatomical complexity, physiological systems, genetics, and cognitive and social behavior [9,10,11,12]. As aging is the major risk factor for synucleinopathies [1], and knowing that these pathologies are progressive, the long lifespan of NHP and the age-dependent accumulation of the neuronal pigment neuromelanin [13,14] are also critical criteria to take into consideration. Even if more costly, NHPs thus provide a higher preclinical model for research in neurosciences. Even if we are aware that studies on other animal models have led to critical advances on synucleinopathies [15,16], we aimed here to present the many NHP models proposed, in chronological order, to study synucleinopathies and to highlight their contribution in understanding the pathogenesis of synucleinopathy (Figure 1).

## 2. Spontaneous Aged-NHP Models

As synucleinopathies are specific to human primates and as aging remains the most compelling and prominent risk factor for the development of synucleinopathies [1], a spontaneous aged NHP profile was explored to model synucleinopathies. In 1998, the study of Emborg and collaborators on young (3–5 years) and aged (26–28 years) rhesus monkeys (*Macaca mulatta*) reported that aged monkeys display motor decline relative to young individuals, with a decrease in general activity, difficulties in achieving fine motor task coordination and the appearance of clinical signs associated with PD profile (bradykinesia, tremor, balance disturbance, stooped posture) [17]. These motor disturbances were associated with a spontaneous reduction of the number of Tyrosine Hydroxylase (TH)-positive neurons and Dopamine Transporter (DAT) immunoreactive cells in the SN. Nevertheless, the magnitude of nigrostriatal dopamine loss observed in this study was insufficient to cause a more prominent parkinsonian syndrome and was proposed as a useful model for human aging and early PD. Although the nigrostriatal dopamine decline in aged NHP was demonstrated here, no report was done regarding the synucleinopathy in aged individuals. In 2007, Chu and Kordower filled the gap and examined the expression of α-syn in individual neurons across a wide range of normal aging NHP [18]. For this purpose, they used rhesus monkeys separated into young (2–12.1 years), middle-aged (15–23 years), and aged (24–34.1 years) groups. Using unbiased stereological counting of nigral α-syn-positive cells, they showed an increase in α-syn-positive neurons by 169% and 215% in the SN of middle-aged and aged monkeys, respectively, compared to young monkeys, demonstrating that accumulation of α-syn in nigral cells was strongly associated with aging. They also found a significant positive correlation between the increase of α-syn immunoreactivity and the decrease in TH immunoreactivity in nigral neurons, suggesting a strong association between the accumulation of α-syn in nigral neurons and the loss of dopamine phenotype. Using proteinase K (PK) treatment, as a readout for the pathological property of α-syn, they reported that age-dependent α-syn accumulation in nigral neurons is soluble and non-aggregated. These data proposed aged rhesus monkeys as a natural and spontaneous model for early PD, given that these animals present a strong increase of soluble α-syn levels associated with a decrease in dopaminergic nigrostriatal phenotype. Using *Microcebus murinus* primates, Canron and collaborators investigated the presence of α-syn in different brain areas of young (1.42–3.07 years) and aged (8.21–10.32 years) animals [19]. They showed that intracytoplasmic α-syn accumulation occurred in the anterior olfactory nucleus, in the cortex, and in the two PD-related regions, SNpc and striatum, of aged but not young monkeys. Interestingly, they observed pathological S129-phosphorylated α-syn in the cerebellum, hippocampus, thalamus, red nucleus, olfactory tubercle, cortex, SNpc, and striatum of old mouse lemur primates. In contrast, no immunostaining was detected in young individuals. Specifically, they noticed that S129-phosphorylated and nitrated α-syn accumulated in the dopaminergic neurons of the SN of aged animals. They thus proposed aged mouse lemurs as a spontaneous model for age-associated α-syn pathology.

In a more recent study, it was also shown that α-syn oligomeric species increase in cytosolic fractions from striatum and hippocampus of middle-aged (10–12 years) and aged (15 years) cynomolgus monkeys (*Macaca fascicularis*) compared to young (3–4 years) individuals [20]. Mitochondrial oligomeric α-syn was demonstrated to increase significantly with age in the striatum, the hippocampus, the cerebellum, and the occipital cortex. Levels of S129-phosphorylated α-syn were also shown to increase according to age, specifically in the striatum and the hippocampus. In 2016, Kimura and collaborators focused on expanding the α-syn profile in normal aging, studying macaque monkeys (Cynomolgus, Japanese, and Rhesus) ranging from 10 to 31 years [21]. They observed a topological pattern of α-syn expansion in the brain according to age, which originated in the midbrain dopaminergic regions such as the SN and the ventral tegmental area, then reached the striatum for the dopaminergic nigrostriatal system and the nucleus accumbens for the dopaminergic mesolimbic system. They also observed an expansion of α-syn occurrence in the mesocortical dopamine system with the appearance of α-syn accumulation in the prefrontal cortex, which increases with age, expanding through motor areas of cortical regions such as the primary motor cortex. They thus demonstrated an age-dependent topological accumulation of α-syn along with the dopaminergic connectivity that can be compatible with the topological expansion of the LB pathology attributed to PD patients. They also noticed the presence of α-syn accumulation in the amygdala and hippocampus, regions related to dementia associated with human DLB pattern. Nevertheless, no LB inclusions or clinical symptoms were observed in aged monkeys. Thereby, they indicated that aged macaques could be useful as a model for studying presymptomatic stages for PD or DLB pathology.

Although dopaminergic neurodegeneration occurs spontaneously according to age in NHP and is associated with accumulation and expansion of α-syn pathology, no LB formation has been observed with aging. Moreover, few of the aged animals used in these studies provide clear clinical phenotypes that resemble human PD, DLB, or MSA pathologies. However, aged NHPs seem to be a relevant model to study normal aging and very early presymptomatic stages of synucleinopathy. Technical difficulties and high costs to allow the NHP to age, and a lack of advanced α-syn pathology considerably decrease the interest in using aged NHP as a spontaneous model for synucleinopathy.

## 3. MPTP-Intoxicated NHP Model

The discovery that 1-methyl-4-phenyl-1,2,3,6-tetrahydropyridine (MPTP) intoxication was able to induce parkinsonism [22] was critical in the development of modeling PD in animals [23]. Acute or chronic MPTP intoxication was shown to induce an oxidative insult through the blockade of the complex I of the mitochondrial respiratory chain by its metabolite, MPP^+^ [24]. This toxic injury specifically induced dopaminergic nigrostriatal lesion associated with motor deficits in different NHP species, including squirrel monkeys (*Saimiri sciureus*) [25], rhesus monkeys (*Macaca mulatta*) [26,27], or marmosets (*Callithrix jacchus*) [28]. For decades, MPTP-intoxicated NHP thus became the gold-standard model to study PD. In 1986, using a chronic protocol to stabilize MPTP intoxication in squirrel monkeys, Forno and collaborators observed a lesion in the locus coeruleus, in addition to the nigral dopaminergic lesion, that provides a supplemental similarity with PD patients’ brains [29]. The similarity with PD brains was also accentuated by the presence of few neuronal eosinophilic bodies in the medulla and nucleus basalis of Meynert in one aged MPTP-intoxicated animal and in the SN and dorsal raphe nuclei in another, which resembled LB inclusions at this time. However, they were less dense and with a weak peripheral halo. In 2000, Kowall and collaborators chose to assess the presence of α-syn pathology in MPTP-intoxicated baboons (*Papio anubis*) [30]. They confirmed the specific dopaminergic lesion in the central region of SN. For the first time, they highlighted a redistribution of α-syn from axons to neuronal cell bodies and dendrites, where they observed α-syn aggregates in the MPTP-intoxicated monkeys compared to the controls. They suggested that oxidative stress induced by MPTP toxin induces aggregation of α-syn as the initial stage of LB inclusion formation. Five years later, Purisai and collaborators showed that α-syn expression was upregulated two to three times, one week after acute MPTP intoxication in aged squirrel monkeys (over 12y) and persisted at one month after intoxication [31]. This upregulation was associated with a redistribution of α-syn protein localization from the neuronal fibers to the cell bodies in the SN one month after MPTP intoxication. Moreover, the number of α-syn-positive neurons in the SN increased in the MPTP-intoxicated animals and was strongly representative of the remaining dopaminergic and neuromelanin-positive neurons. This work highlighted two different directions in the relationship between α-syn accumulation and dopaminergic lesions. The authors first proposed that long-term lasting α-syn upregulation was triggered solely by an acute MPTP insult and might participate in dopaminergic neuronal cell loss, suggesting a toxic gain-of-function of α-syn in the pathology. On the other hand, as the increase of α-syn cells occurred in the surviving dopaminergic neurons, the data highlighted a compensatory response of neuronal cells to toxic injury. They also concluded that these two mechanisms could act at different timepoints in the pathology with a first α-syn response to protect dopaminergic neurons that were reversed into pathological mechanisms by other undetermined factors. The same team went further and focused on the induced synucleinopathy characteristics after acute MPTP injury in middle-aged squirrel monkeys [32]. They confirmed that MPTP insult induced α-syn upregulation and nigral dopaminergic cell loss one-month post-intoxication. They also highlighted that the increase in α-syn immunoreactivity was specifically neuronal. No α-syn was localized in glial cells in MPTP-intoxicated animals, suggesting that MPTP-monkeys could not be used as a MSA pathology model [32]. For the first time, they showed that nitrated and S129-phosphorylated α-syn accumulated in neuromelanin-containing neurons and dystrophic neurites. They also demonstrated that neuronal α-syn was soluble using PK treatment, whereas 15% of neuritic α-syn were PK-resistant and possibly aggregated. These data suggested that the MPTP insult induced oxidative stress that might lead to α-syn modifications such as nitration and phosphorylation, which could be key events in the aggregative process of α-syn observed in damaged axons. They hypothesized that the accumulation of aggregated α-syn in damaged axons might dysregulate the normal synaptic distribution of α-syn protein and contribute to its accumulation in dopaminergic neuronal cell bodies as the initial stage of LB formation. Nevertheless, no LB were observed in the MPTP-intoxicated monkey. These data also pointed out the importance of age in developing α-syn pathology induced by MPTP insult on NHP. To test the hypothesis that LB formation required a long period after MPTP intoxication to be initiated, Halliday and collaborators assessed the α-syn-related pathology presented in middle-aged cynomolgus monkeys (*Macaca fascicularis*) treated ten years before chronically with MPTP for over two years [33]. Animals presented sustainable parkinsonism lasting for ten years, associated with a strong dopaminergic neuronal loss and increased intraneuronal α-syn and S129-phosphorylated α-syn, which accumulated in the remaining nigral neurons [33]. Nevertheless, LB were still not observed in these animals. This work highlighted that, in addition to the length of intoxication, the age of the intoxicated animal must also play a role in the severity of the α-syn-related pathology. In 2018, Huang and collaborators adopted a specific MPTP modeling-recovery-MPTP remodeling strategy on aged MPTP-intoxicated rhesus macaques (17 and 21 years) to stabilize parkinsonian syndrome without any recovery of the animals [34]. They found a significant negative correlation between the number of nigral TH-positive cells and S129-phosphorylated α-syn aggregation in the SN of these aged MPTP monkeys. These data confirmed that using MPTP intoxication in aged monkeys is a relevant new model to understand mechanisms underlying α-syn-related pathology. Using a large cohort of young rhesus monkeys (*Macaca mulatta*) chronically treated with MPTP, it was recently shown that α-syn-related pathology was not only present in the dopaminergic neurons of the SN but widespread to motor-related structures, such as the putamen, and to non-motor cortical regions, such as the hippocampal CA1 and the temporal cortex [35]. More interestingly, by treating some MPTP animals with the gold-standard pharmacological treatment L-DOPA, they demonstrated that L-DOPA abolished the MPTP-induced α-syn accumulation in the putamen and the cortical areas and decreased the amount of α-syn-positive neurons observed in the SN. These data highlighted the relevance of employing MPTP-intoxicated NHP in a translational approach that aimed to halt MPTP induced-α-syn pathology. Finally, it has been shown that MPTP-intoxicated monkeys can be useful to study peripheric α-syn pathology associated with PD. Human autopsy studies have consistently shown that LB are found in the ENS in nearly every case examined [36]. In 2020, Li and colleagues showed that total, S129-phosphorylated and oligomeric α-syn amounts are increased in the SN and the striatum as well as in the colon of middle-aged MPTP-intoxicated cynomolgus monkeys (10–12 years) [37]. They also observed dopaminergic neurodegeneration in the ENS and alterations in the expression of two enzymes involved in α-syn phosphorylation/dephosphorylation. Interestingly, they did not only report for the first-time development of α-syn pathology in the ENS of MPTP-intoxicated NHP but also found a positive correlation between levels of α-syn in the CNS and ENS. As they studied the tissues at only one-month post-intoxication, the α-syn pathology and dopaminergic lesion present in the CNS and ENS resulted from independent local response to intravenous MPTP injection and not a consequence of a long-distance propagation of α-syn pathology. Nevertheless, this work demonstrated the relevance of the MPTP NHP model to bring new information in an attempt to understand the mechanisms underlying central and peripheric PD pathogenesis.

Using MPTP-intoxicated monkeys, these studies first pointed out the role of a potential environmental and external toxic insult in the induction of neurodegeneration and α-syn pathology, two important characteristics involved in the pathogenesis of synucleinopathy. Specifically, they showed that MPTP increases oxidative stress through the blockade of the complex I of the mitochondrial respiratory chain by its metabolite MPP+ and modifies α-syn expression and protein localization, and promotes pathological α-syn aggregation. Notably, age and duration of intoxication with MPTP in NHPs were shown to be the key players to obtain an experimental model with a stable clinical pattern associated with dopaminergic neurodegeneration and α-syn-related pathology. Thus, aged and intoxicated NHP could represent a relevant model to study the synucleinopathy, recapitulating two important criteria to model in particular PD, with a specific dopaminergic lesion and a widespread neuronal accumulation of pathological α-syn forms. Nevertheless, no advanced LB-like pathology was observed in the MPTP-intoxicated monkey, despite a clear and stable clinical parkinsonian syndrome associated with a robust dopaminergic lesion. In the future, the MPTP modeling-recovery-MPTP remodeling strategy adopted by Huang and al [34] should be considered in middle-aged monkeys to enable a safe and stable induction of parkinsonian motor syndrome after a long-term period of intoxication, as well as strong neurodegeneration that might be associated with advanced synucleinopathy, including LB formation. Nevertheless, the high toxic sensitivity of old monkeys to MPTP and the technical and pecuniary issues to let the monkeys age considerably slow down the interest in using aged MPTP-intoxicated monkeys as a model for synucleinopathies.

## 4. Viral Vector-Mediated Models

With the development and fine-tuning of viral production, certain studies have aimed at injecting viral vectors to overexpress either wild-type (WT) or mutant α-syn to induce pathology in NHPs. The goal of these viral vector-mediated models is to replicate more closely the pathology in NHP, mainly by causing not only loss of dopaminergic neurons, but also an accumulation of α-syn in neurons or oligodendrocytes. Besides, this viral-mediated overexpression has the advantage of targeting specific regions or cell types in the brain, making their use of interest in the study of all synucleinopathies.

### 4.1. Viral Vector-Based Models of Parkinson’s Disease

Intracerebral injections of adeno-associated viruses (AAV) were first validated in rodents, particularly in rats, before being tested in NHP [38,39]. Building on their study in rats, Kirik and colleagues injected AAV1/2 to overexpress GFP, WT-α-syn, or A53T-mutant α-syn unilaterally in neurons in the right SN of common marmosets [40]. Three weeks post-injection, they verified their AAV’s correct expression in control (GFP) monkeys and found that their virus was indeed specifically expressed in neurons. Following this, they waited four months post-injection to assess their various AAV effects on neurodegeneration and α-syn. In A53T-α-syn marmosets, they observed a loss of TH immunostaining in the SNpc with a decrease in VMAT-2. They also observed the presence of TH-positive fragmented neurites and α-syn-positive inclusions. This was accompanied by behavioral changes with a bias in the head position test on the ipsilateral side, starting at six weeks post-injection. This study was followed by using AAV2/5, also under the same neuronal promoter, targeting GFP, WT-α-syn, and A53T-mutant α-syn in a larger group of common marmosets [41]. The long-term effects of these unilateral injections in the SN were assessed one year after the AAVs injection. During the one-year live phase, they observed contralesional motor deficits in A53T-α-syn injected monkeys, with worsening general motor coordination over time. Post-mortem analysis showed that these monkeys displayed loss of TH immunostaining in the SN of WT and A53T-α-syn injected monkeys. They also observed total and S129-phosphorylated α-syn-positive inclusions, with the added presence of ubiquitin in these inclusions in the A53T-injected group. Both of these studies showed that both WT and A53T-mutant α-syn had deleterious effects when overexpressed in marmosets, but the A53T-mutant was more potent in inducing a PD-like pathology. These two first studies in marmoset monkeys were essential in demonstrating the ability to overexpress α-syn via viral vectors in NHPs, leading to decreased dopaminergic neurons and the formation of α-syn inclusions, associated with motor behavior.

Other approaches have consisted of injecting lentiviral vectors to force the expression of A53T-mutant α-syn in the SN of rhesus monkeys [42]. Yang and colleagues wanted to assess both the effect of injecting lentiviral vectors containing mutant α-syn and if this effect depended on the monkeys’ age. After first verifying their lentivirus’s correct expression in control and A53T-injected monkeys, they observed the initial formation of small α-syn aggregates in long neuronal processes, similar to Lewy neurites, in the A53T-injected monkey. They next injected monkeys of different ages (2y, 8y, and over 15y) with either PBS or the A53T lentivirus. After eight weeks, A53T monkeys demonstrated the formation of Lewy neurites and astroglial activation, which were more abundant in older monkeys than in younger monkeys. These monkeys also presented axonal degeneration and TH immunostaining loss in the SN, specifically in A53T-injected monkeys. As previously stated, age has been shown to impact the accumulation of α-syn in the brain. This study demonstrated that monkeys’ age plays a role in neuropathology when combined with lentiviral overexpression of A53T-α-syn.

Similarly, Bourdenx and colleagues wanted to assess whether age was a factor in developing PD neuropathology. For this purpose, they injected an AAV2/9 to overexpress the A53T-α-syn mutant using a neuronal promoter in the SN of young and old marmoset monkeys [43]. Eleven weeks post-injection, they first observed decreased TH immunostaining in sham-operated old animals in the SN and striatum. They observed a decrease in TH immunostaining in the SN and the striatum in both young and aged monkeys in the injected side. Concerning α-syn pathology, both young and old monkeys demonstrated an increase in total and S129-phosphorylated α-syn. Surprisingly, old monkeys presented less α-syn phosphorylation than young animals. Here, they showed that overexpression of α-syn induced a decrease in dopaminergic neurons and fibers accompanied by α-syn accumulation. Still, the age of monkeys did not impact this pathological progression. These last two studies aiming at determining the part of age in α-syn accumulation have shown quite diverging results. On the one hand, Yang and colleagues demonstrated that A53T overexpression impacts older monkeys more severely, while Bourdenx and colleagues did not observe this same effect of age. This could be due to the difference in species used, with one study using rhesus monkeys and the other using common marmosets or lentivirus compared to AAV.

More recently, Koprich and colleagues injected AAV1/2 A53T-α-syn in the SN of cynomolgus macaques [44]. In this study, they used different parameters to determine the conditions in which sustained expression of α-syn would induce neurodegeneration. In a first experiment, when injecting the virus at four sites of the SN, no dopaminergic neuron loss was observed. To optimize their AAV effects, they used higher titers or larger volumes in their second experiment. Both high titers and larger volumes were able to induce the loss of dopaminergic neurons in the SN. Nonetheless, the injection of a higher titer of virus had a more substantial effect on the decrease of DAT and dopamine than the injection of larger volumes. On the contrary, injecting larger volumes of virus induced higher levels of putaminal α-syn. Altogether, this study showed the impact of both the titer and volume of injection in monkeys, demonstrating once more that diverging results between experiments could not only be due to the species used but also to the injection itself.

### 4.2. Viral Vector-Based Models of Multiple System Atrophy

Given the promising studies using viral vectors to induce PD pathology, certain studies aimed to use viral vectors to generate other synucleinopathies, and more specifically, MSA. In mice, several studies have successfully overexpressed α-syn specifically in oligodendrocytes and recapitulated certain aspects of the neuropathology, including some motor deficits [45,46,47]. In a first study, Bassil and colleagues injected AAV1/2 with α-syn driven by an oligodendroglial promoter in both rats and macaque monkeys [48]. After putaminal injection of α-syn, they demonstrated specific α-syn localization in oligodendrocytes, with little neuronal expression of α-syn, highlighting that such an AAV-mediated approach may be usable in NHP. This study provided the first description of AAV-mediated transgene expression in oligodendrocytes in the NHP that was achieved using a cellular promoter. Nonetheless, further longitudinal studies will determine the progression of behavioral deficits and MSA-like pathology in this species. Concomitantly, Mandel and collaborators injected AAV-Olig001 targeting either GFP or α-syn in oligodendrocytes of rhesus monkeys [49]. The authors first demonstrated the control virus’s efficacy to express GFP in oligodendrocytes of the striatum after four weeks. Three months post-injection in the striatum, they observed α-syn and S129-phosphorylated α-syn aggregates in the caudate nucleus, the putamen, and corpus callosum of α-syn-injected monkeys. These phosphorylated inclusions were shown to be PK resistant, demonstrating the production of insoluble aggregates. Besides, they established that these monkeys showed demyelination of the corpus callosum combined with microglial activation in the striatum. Following this study, Marmion and colleagues also wanted to determine the effect of high titer AAV-Olig001 targeting α-syn in cynomolgus macaques six months after injection [50]. α-Syn was shown to accumulate in the putamen and to display inclusions that were α-syn-positive and S129-phosphorylated, and Y39-phosphorylated-α-syn-positive. Given this, they concluded that AAV-Olig001-α-syn was able to induce the formation of GCI-like inclusions in macaques. These monkeys also showed neurodegeneration with decreased TH immunostaining in the SN and demyelination in the striatum. Altogether, they demonstrated that injections with their viral-vector induced an MSA-like pathology in macaques with the formation of GCI-like inclusions, neurodegeneration, demyelination, and inflammation.

Taken together, these studies proved that the use of viral vectors was able to induce a sustained expression of α-syn in the SN of various monkeys, including marmosets and macaques. Despite the multiple viruses and serotypes, driving promoters, and injection periods tested, they were able to observe sustained α-syn overexpression accompanied, for the most part, by a loss of dopaminergic neurons in the SN. Injection of viral vectors demonstrated for the first time that, by using a cell-specific promotor overexpressing α-syn, it is possible to create models for different synucleinopathies. In certain studies, these viral-vectors were combined with the use of older monkeys to establish models closer to human conditions. Nonetheless, these viral vectors have yet to recapitulate all aspects of synucleinopathies but are of high interest in the study of these diseases in NHP.

## 5. Patient-Derived Brain Extracts Models

In another way to model synucleinopathies, we and other groups have used patient brain-derived material to get closer to the human pathophysiology in terms of the nature of inoculated material and the amount of injected α-syn. In 2014, Recasens and collaborators used LB-enriched fractions purified from three PD patients’ mesencephala, which contained amyloid-like structures and insoluble aggregated α-syn, to inject ng of pathological α-syn into mice and NHP [51]. They showed that injection of LB-enriched fractions into mice induced significant dopaminergic neurodegeneration, accompanied by accumulation of PK-resistant and S129 phosphorylated forms of endogenous α-syn in the SN, the striatum, and the neocortical areas. These data were the first proof of concept regarding the induction of the pathogenicity from the human pathogenic material towards the endogenous murine α-syn protein. Along with the demonstrated aggregation and propagation of α-syn into interconnected brain areas, this study brought strong evidence about the “prion-like” hypothesis of α-syn in synucleinopathies [52]. More interestingly, LB-derived fractions into either the SN or the striatum were injected into four rhesus monkeys (8y). One monkey of each group was previously chronically treated with MPTP three years before. Using PET imaging, they demonstrated that striatal and nigral LB-inoculated monkeys exhibit a striatal dopaminergic lesion that appeared at nine months and lasted up to twelve months. Fourteen months after the LB inoculation, they obtained a more pronounced dopaminergic cell loss in the SN of striatal-injected monkeys than the nigral-inoculated animals. They also showed that, in striatal LB-inoculated primates, PD-derived LB extracts induced a widespread increase of α-syn levels in interconnected regions suggesting a long-distance propagation of α-syn pathology, which appeared to be more local for the nigral-injected groups. Interestingly, striatal inoculation of LB fractions in MPTP-treated monkeys did not lead to α-syn pathology in the SN but instead to an aggravated increase of α-syn into striatal and efferent areas, suggesting a retrograde transmission of α-syn from the striatum to the SN. This work was the first proof-of-concept showing that LB extracts purified from patients’ brains induced a pathological response in NHP, including neurodegeneration and a “prion-like” synucleinopathy. To confirm this result, a follow-up study aimed at investigating the consequences of PD-derived LB inocula on a larger cohort of NHP. The authors injected PD patient-derived LB fractions containing large and insoluble α-syn aggregates in the striatum of olive baboons (*Papio papio*) [53]. Two years after the injection, LB-inoculated NHP present nigrostriatal neurodegeneration associated with α-syn pathology localized in different brain regions. More surprisingly, and in contrast with mice, when PD patient-derived noLB fractions, containing small aggregates and mainly soluble α-syn, were injected into NHP, they observed dopaminergic neurodegeneration to the extent of LB-inoculated monkeys, also associated with α-syn pathology localized in many brain regions. Taking advantage of a machine learning approach, the authors sorted out the twenty variables that constituted the best predictors of neurodegeneration among a dataset of 180 measured variables for the two injection groups. Interestingly, they obtained unique pathological signatures of induced pathology between LB and noLB groups, leading to the same dopaminergic lesion level. This study showed that distinct pathological α-syn species led to the same dopaminergic lesion level through different underlying mechanisms, modeling the multifactorial nature and complexity of synucleinopathies. Using the same type of patient brain-derived extracts, the same authors decided to inject LB fractions in both the stomach and ventral duodenum wall of five baboon monkeys. The underlying idea was to compare the pathology induced with the one obtained for the striatal LB-fraction injected group and to challenge the hypothesis of a caudo-rostral propagation of α-syn pathology presumed by Braak and colleagues [54]. Interestingly, they observed that enteric inoculation of LB fractions in NHP led to central dopaminergic neurodegeneration in the SN and the striatum, at the same level as for the striatal-LB-injected group, associated with the development of α-syn pathology in the CNS. These data suggested that α-syn pathology propagated in a caudo-rostral fashion from the ENS towards the CNS. More surprisingly, they found that not only did the enteric LB-injected animals induce a local α-syn accumulation in enteric neurons, but so did the striatal-LB-injected animals. Moreover, they observed a significant negative correlation between the number of nigral dopaminergic neurons in the CNS and the amount of α-syn in the ENS neurons, suggesting that the enteric α-syn pathology extent may reflect the severity of the central dopaminergic lesion. Of interest, these data demonstrated that α-syn pathology also propagated rostro-caudally from the CNS towards the ENS. Regarding the bidirectional routes of propagation of the synucleinopathy, the vagus nerve was put aside in this experimental model, and biological fluids have been considered as possible alternative routes of α-syn pathology spreading.

Injection of LB-enriched fractions in NHPs recapitulates two important neuropathological criteria to model synucleinopathies: neurodegeneration and presence and spreading of α-syn pathology. Hence, LB-enriched inoculation in NHP may provide a relevant model to better understand the mechanisms underlying the pathology in synucleinopathy. However, only subtle behavioral changes assessed by validated ethological evaluation have been observed two years after the injection of LB-fraction, due to a dopaminergic cell loss that does not reach the threshold of the appearance of the motor symptoms. This progressive model can thus be employed to mimic the early stages of synucleinopathies to decipher the underlying mechanisms and better understand the “prion-like” properties of α-syn. Assessing α-syn pathology and neurodegeneration at early timepoints after LB injection has to be performed to evaluate the pathological signature dynamics of the pathology at very early stages. Similarly, increasing LB post-injection duration could be considered to assess a more advanced picture of neurodegeneration and α-syn pathology. Finally, inoculating other types of pathogenic brain extracts such as GCI fractions derived from MSA patients could be envisaged trying to model the other synucleinopathies and mimic their own specificity. Limited access to human material remains the drawback of using NHP models based upon human brain-derived extracts. Considerable collective efforts have to be made to find innovative solutions to bypass the need for fresh human material.

## 6. Recombinant α-Syn Preformed Fibrils Models

Injections of α-syn preformed fibrils (PFFs) in vitro and in mice have shown their efficacy in inducing both loss of dopaminergic neurons of the SN and the formation of aggregates of insoluble α-syn [55,56,57]. Following the demonstration of this in rodents, Shimozawa and collaborators endeavored to determine whether this was also the case in marmoset monkeys [58]. For this purpose, they injected marmoset monkeys with mouse recombinant α-syn fibrils in the caudate nucleus and putamen. Three months after injection, they observed abundant S129-phosphorylated α-syn structures throughout various brain regions, including the striatum, SN, cortex, amygdala, thalamus. The inclusions seen were also positive for human α-syn, ubiquitin, and p62 staining. Formation of round, LB-like, S129-phosphorylated α-syn-positive inclusions was detected in dopaminergic neurons three months post-injection. Neurodegeneration was also observed with the decrease of TH-positive staining in the SN. Colocalization of the human-specific antibody LB509 and the microglial marker Iba1 suggested that the inclusions were phagocytosed by microglial cells. Altogether, this study showed for the first time that the use of PFFs could induce the formation of LB-like inclusions and dopaminergic neurodegeneration in the SN of marmoset monkeys. More recently, Chu and colleagues used injections of PFFs in the striatum of macaque monkeys to determine the effects of human recombinant α-syn fibrils [59,60]. Cynomolgus monkeys received intrastriatal injections of PFFs, and four control monkeys received sham surgery. After 12 to 15 months, they observed loss of TH immunostaining, accompanied by increased striatal DAT immunostaining. In addition, they observed S129-phosphorylated-α-syn inclusions in the SN that demonstrated two different aspects: either a more granular aspect or a whole-cell staining with absent cytoplasm, similar to the beginning of LB formation. These nigral neurons containing α-syn inclusions had lost both their TH and Nurr1 staining, reminiscent of PD pathology. In both cases, these aggregates suggested progressive α-syn accumulation, leading to the formation of larger LB-like inclusions. Both of these studies have shown the possibility of inducing a PD-like pathology in both marmoset and macaque monkeys. Despite using mouse or human α-syn PFFs, these studies demonstrated the ability of α-syn propagation and aggregate formation in NHPs. Nonetheless, it is important to note that large quantities of α-syn fibrils had to be injected to induce these PD-like pathologies. These are not sufficient to induce the appearance of clinical symptoms. Recently, other PFF delivery methods have been tested in cynomolgus monkeys by using intranasal injections [61]. After either one, four, or seventeen months, Guo and colleagues observed an accumulation of iron, accompanied by a sparse appearance of S129-phosphorylated α-syn, which did not colocalize with iron deposits. Nonetheless, this type of administration of PFFs did not induce dopaminergic neurodegeneration. Altogether, these studies showed the attempt of growing use of PFFs in NHP models and their potential, depending on their administration, to cause a PD-like pathology.

## 7. Transgenic Models

The purpose of a transgenic NHP synucleinopathy model would be to have a progressive and universal model able to recapitulate aspects of the disease from birth. Given the constraints, ethical and costly, and technical challenges, even with the recent progress in transgenesis, very few studies have attempted to create transgenic synucleinopathy models. In 2014, Niu and colleagues endeavored to generate a transgenic monkey model by lentiviral vector injection in fertilized monkey eggs [62]. After expression of A53T-mutant α-syn in oocytes, 75 eggs were transferred in rhesus monkeys. These transfers resulted in 11 pregnancies and led to the birth of live newborn monkeys. Transgene expression was confirmed, and immunostaining also showed the presence of increased α-syn in the SN, striatum, and cortex, but not of S129-phosphorylated α-syn in these A53T-transgenic monkeys. The authors also noted that older monkeys developed cognitive defects and anxiety starting at 2.5 years of age. These defects implicated object recognition, dexterity, and stereotypical circling behavior of these animals, reminiscent of prodromal defects seen in PD patients. Nonetheless, these animals showed no motor abnormalities and no neurodegeneration using MRI. Compared to other models, these transgenic monkeys could be more reliable to observe phenotypes and pathological modifications and find biomarkers for PD. Given that this study only followed the A53T-transgenic monkeys for 2.5 years, it is difficult to say whether they will develop other aspects of PD in the future. Still, this study remained highly interested in observing the age-dependent factors of PD onset. More recently, with the discovery of CRISPR-Cas9 technology, certain studies have taken advantage of the mere use of this system to develop transgenic models of synucleinopathy. Yang and colleagues injected CRISPR-Cas9 directed against the *PINK1* gene in one-cell stage embryos from rhesus monkeys [63]. After the transfer of the embryos to surrogate rhesus monkeys, eleven fetuses developed (8 *PINK1* mutants and 3 WT) and were born naturally. Of these, four mutant and one WT monkey died in the first week after birth. Of the four remaining mutant monkeys, one lived for 1.5 years, and the three others were terminated three years after birth. Certain monkeys with *PINK1* mutations displayed decreased grey matter density in the cortex, and others had decreased movement after 1.5 years. After 1.5 years, decreased neuronal immunostaining and increased astrogliosis compared to WT monkeys were observed. Electron microscopy in one mutant monkey demonstrated degeneration of neurons in the cortex, SN, and striatum. Despite not demonstrating any modifications in α-syn or its distribution, this team showed the possible use of CRISPR-Cas9 relating to PD in NHPs. Regardless of the uncommon use of transgenic NHP models in PD, it is important to note their potential importance for future studies. Given the constraints observed, it is not surprising that few studies currently exist that have created transgenic NHPs. Still, we suspect that the number of studies will grow in the next years to resolve the need to better understand intractable diseases such as PD.

## 8. Conclusions

Here, we have discussed the existing NHP models of synucleinopathy that have been developed and emerged in the last years. Different approaches have been used among these models, such as natural aging of animals and injections of toxins, viruses, or α-syn-based products (summarized in Table 1). Together, these NHP models present their advantages and limits, noting that most models lack certain aspects found in human pathologies [16]. The central difficulty has been observing the formation of LB, as can be found in the human pathology, with the formation of dense bodies with a peripheral halo. Given the many monkey species available and the cost they engender, the species is not always an easy choice but can play a significant role in the results observed. As seen previously, the same type of models can have different results based on the species used, particularly between New World and Old World monkeys. New World monkeys present specific differences in their α-syn sequence, including the natural appearance of the A53T mutation, which the Old World monkeys do not show [64]. Besides, the presence of neuromelanin has been detected in Old World monkeys, but not in New World monkeys, which could explain specific differential susceptibilities between species [14,65,66]. These differences between Old World and New World monkeys have been thoroughly previously discussed, along with the impact of genetic modifications in NHP models [67]. Furthermore, it is important to note that the lifespan between monkey species can differ tremendously, making the study of aging difficult. For example, marmoset monkeys live around ten years, whereas macaque monkeys live around 25–30 years [68]. Even taking all these aspects into account, the variability between injections and quantities injected, this remains difficult to compare two experimental models side by side. Moreover, NHP models render substantial costs related to the animals’ maintenance and care and the difficulties of having a sufficient number of monkeys. Nonetheless, many models have shown interesting findings in NHP that mimic more closely than in rodents, the appearance of certain neuropathological features. This could be mainly due to the closer anatomical resemblance of monkeys to humans, compared to rodents. In WT rodents, no age-dependent accumulation of the neuronal pigment neuromelanin occurred. Furthermore, the α-syn protein sequence diverged between rodents and primates, with, for instance, the natural presence of the A53T mutation in rodents. It is equally important to note that other animal models, though less ethically challenging, have been unable to show such similarities with human synucleinopathy than NHP models. Among the NHP models of synucleinopathy, some teams aim to combine both aging and intracerebral injections to obtain a model closer to human PD. In some cases, this approach has successfully demonstrated the more important effect of injections on aged animals [32,42], while in others, no such differences were observed [43]. Despite these variable effects of age, this could be an interesting strategy to study synucleinopathies in the future.

Since the discovery of its implication in LB formation, α-syn has been very much at the center of most PD and MSA models. Studies have been carried out to elucidate its ability to be a trigger, a biomarker, and/or a therapeutic target for PD. In various models, α-syn overexpression induces not only dopaminergic cell loss in the SN, but also demonstrates its ability to propagate and seed the formation of new α-syn aggregates. The main foundation of α-syn overexpressing models is based on the presence of PD patients presenting genetic duplication or triplication of the *SNCA* gene [69,70]. Although only certain of the abovementioned models use this overexpression to induce PD pathology, most recent models demonstrate α-syn accumulation in their studies despite their lack of induced α-syn overexpression. Overall, current studies have mostly focused on PD NHP models, with very few studying DLB or MSA. In the future, additional efforts should also be placed on these two other synucleinopathies to create models that recapitulate their cell-specific α-syn accumulation and neuropathology. The nature of α-syn assemblies (i.e., oligomers, fibrils) used for intracerebral injection may be the source of variabilities at different levels mainly and not exclusively due to (i) α-synuclein inoculum preparation (protein folding, protein concentration, the composition of the vehicle, detergent-insoluble fractions vs. total brain homogenates vs. LB-bearing fractions); (ii) α-synuclein inoculum purity (molecular size, sonication protocol, and storage conditions); (iii) volume of injected material and speed of injection; and (iv) time post-injection. Altogether, the resulting anatomopathological features will be dependent per se from the injected material, and in its ability to form oligomers and/or fibrils, to self-maintain and propagate over-time in vivo. It is equally important to note that no current consensus exists that defines the observation and the criteria of synucleinopathy occurrence in models [71]. Many studies observe various aspects of α-syn accumulation in their respective studies, whether it is the presence of PK-resistant α-syn, S129-phosphorylated α-syn, or distinct α-syn conformers. Better terms and standardized methods to characterize α-syn are eagerly awaited in the field. Overall, it would be essential to agree upon certain aspects that must be demonstrated before validating an adequate model to study these synucleinopathies.

Transgenic NHP models are one of the methods that have yet to be thoroughly studied, given the complications of such experiments. Despite this, studies have shown the potential interest of these models to have a progressive installment of the disease, without requiring injections. In fact, other neurodegenerative disorders such as Huntington’s Disease have generated transgenic monkey models to recapitulate main aspects of diseases [72,73]. This transgenic monkey model shows a progressive installment of the disease, with the presence and progression of aggregated huntingtin protein throughout the brain, and progressive striatal atrophy. With the limited current data on α-syn transgenic monkeys, it has yet to be demonstrated whether there will be a valid model and, more importantly, how long the disease onset will require. It is important to note that new strategies using CRISPR-Cas9 technologies should prove to be assets in developing such NHP models [74].

Despite the lack of models that recreate the full spectrum of synucleinopathy as in PD or MSA patients, NHP models have come further in recapitulating the majority of neuropathological aspects of these diseases. Together and to date, these multiple NHP models demonstrated the capacity of inducing the early stages of synucleinopathy through different approaches. Understanding these early stages of α-syn aggregation and neurodegeneration could be extremely important in having a better comprehension of the establishment of PD, DLB, and MSA. With the improvement of strategies for developing models such as the injection of viral vectors, patient-derived extracts, preformed fibrils, we have been seeing a new rise in NHP models of synucleinopathy, which should continue to progress in the next decade.

## Figures and Tables

**Table 1:** Summary of non-human primate models of synucleinopathy.

Type of Model	NHP Species	Injection	Pathological Phenotype	Reference
Aging	Rhesus monkeys (young: 3–5y; aged: 26–28y)	NA	Clinical motor symptoms Dopaminergic cell loss (aged animals)	[17]
	Rhesus monkeys (young: 2–12y; middle-aged: 15–23y; aged: 24–34.1y)	NA	Dopaminergic neurodegeneration α-syn accumulation in the SN (middle-aged and aged animals)	[18]
	Mouse lemur primates (*Microcebus murinus*) (young: 1.42–3.07y; aged: 8.21–10.32y)	NA	α-syn accumulation in anterior olfactory nucleus, cortex, SN, and striatum S129-phosphorylated α-syn in the cerebellum, hippocampus, thalamus, red nucleus, olfactory tubercle, cortex, SNpc, and striatum (aged animals)	[19]
	Cynomolgus monkeys (*Macaca fascicularis*) (young: 3–4y; middle-aged: 10–12y; aged: 15y)	NA	Accumulation of oligomeric and S129-phosphorylated α-syn in striatum and hippocampus (middle-aged and aged animals)	[20]
	Macaques (Cynomolgus, Japanese, and Rhesus) (10–31y)	NA	Accumulation of α-syn in the SN, ventral tegmental, striatum, nucleus accumbens, prefrontal cortex, primary motor cortex	[21]
MPTP	Squirrel monkeys (*Saimiri sciureus*) (young: 5–10y; old:15–20y)	Chronic MPTP	Clinical parkinsonism Dopaminergic neurodegeneration Neuronal eosinophilic bodies	[29]
	Baboon (*Papio anubis*)	Chronic MPTP	Clinical parkinsonism Dopaminergic neurodegeneration Redistribution of α-syn from axons to cell bodies	[30]
	Squirrel monkeys (*Saimiri sciureus*) (aged: >12y)	Acute MPTP	Clinical parkinsonism Dopaminergic neurodegeneration α-syn upregulation and accumulation in the SN	[31]
	Squirrel monkeys (*Saimiri sciureus*) (aged: >12y)	Acute MPTP	Clinical parkinsonism Dopaminergic neurodegeneration S129-phosphorylated, nitrated and PK-resistant α-syn in the SN	[32]
	Cynomolgus monkeys (*Macaca fascicularis*) (14y)	Chronic MPTP	Clinical parkinsonism Dopaminergic neurodegeneration S129-phosphorylated α-syn accumulation in the SN	[33]
	Rhesus monkeys (*Macaca mulatta*) (17–21y)	MPTP modeling-recovery-MPTP remodeling	Clinical parkinsonism Dopaminergic neurodegeneration S129-phosphorylated α-syn accumulation in the SN	[34]
	Rhesus monkeys (*Macaca mulatta*) (5.4 ± 1y)	Chronic MPTP	Clinical parkinsonism Dopaminergic neurodegeneration α-syn accumulation in the SN, putamen, and cortical areas	[35]
	Cynomolgus monkeys (*Macaca fascicularis*) (10–12y)	Chronic MPTP in the CNS and ENS	Clinical parkinsonism Dopaminergic neurodegeneration in the CNS and ENS S129-phosphorylated and oligomeric α-syn accumulation in CNS and ENS	[37]
Viral vectors	Common marmoset (*Callithrix jacchus*) (5.5–6y)	AAV1/2-CBA GFP, A53T-α-syn or WT-α-syn (4 months)	Behavioral changes: head position bias starting at 6 weeks Dopaminergic neurodegeneration α-syn-positive inclusions	[40]
	Common marmoset (*Callithrix jacchus*) (5.5–6y)	AAV2/5-CBA GFP, A53T-α-syn or WT-α-syn (12 months)	Behavioral changes: contralesional motor deficits in A53T-α-syn monkeys + worsening general motor coordination Dopaminergic neurodegeneration α-syn and S129-phosphorylated α-syn inclusions in A53T group	[41]
	Rhesus monkeys (young: 2y; middle-aged: 8y; old: 22y)	Lentiviral A53T-α-syn (2months)	Axonal damage and dopaminergic neurodegeneration Accumulation of α-syn (particularly in older monkeys) Increased astroglial activation	[42]
	Common marmoset (*Callithrix jacchus*) (young: 2y; old: 5y)	AAV2/9-CMVie A53T-α-syn (11 weeks)	Dopaminergic neurodegeneration Accumulation of α-syn and S129-phosphorylated α-syn	[43]
	Cynomolgus monkeys (*Macaca fascicularis*) (8y)	AAV1/2-CBA/CMV A53T-α-syn High titer/low volume or low titer/large volume (4 months)	Dopaminergic neurodegeneration Higher α-syn levels by ELISA	[44]
	Rhesus monkeys (*Macaca mulatta*)	AAV-Olig001-α-syn (3 months)	Soluble and aggregated α-syn observed in the caudate and nucleus Formation of GCI-like inclusions Demyelination and microglial activation	[49]
	Cynomolgus monkeys (*Macaca fascicularis*)	AAV-Olig001-α-syn (6 months)	Dopaminergic neurodegeneration α-syn-positive GCI formation (pS129+, pY39+, LB509+) Demyelination and microglial activation	[50]
Patient brain-derived extracts	Rhesus monkeys (*Macaca fascicularis*)	PD brain-derived LB fraction (14 months)	Dopaminergic neurodegeneration α-syn accumulation and propagation	[51]
	Olive baboon (*Papio papio*)	PD brain-derived LB fraction PD brain-derived noLB fraction (24 months)	Dopaminergic neurodegeneration α-syn accumulation and propagation Distinct pathogenic signature	[53]
	Olive baboon (*Papio papio*)	PD brain-derived LB fraction in the CNS and ENS (24 months)	Dopaminergic neurodegeneration (CNS) α-syn accumulation and propagation (CNS and ENS) Bidirectional propagation of α-syn pathology	[54]
Preformed fibrils	Common marmoset (*Callithrix jacchus*) (2y)	Mouse recombinant α-syn fibrils (3 months)	Dopaminergic neurodegeneration S129-phosphorylated α-syn in the striatum, SN, cortex, amygdala, thalamus, and others Formation of LB-like structures	[58]
	Cynomolgus monkeys (*Macaca fascicularis*) (6–10y)	Human recombinant α-syn fibrils (12–15 months)	Dopaminergic neurodegeneration Increase in DAT staining S129-phosphorylated α-syn inclusions in the SN (granular and whole-cell inclusions) Loss of Nurr1 and TH staining in α-syn-positive inclusions	[59]
	Cynomolgus monkeys (*Macaca fascicularis*) (6–10y)	Human recombinant α-syn fibrils (1, 4, 17 months)	Accumulation of iron in microglia Weak S129-phosphorylated α-syn immunostaining No dopaminergic neurodegeneration	[61]
Transgenic models	Rhesus monkeys (*Macaca fascicularis*)	Lentiviral A53T α-syn in oocytes	Cognitive defects and anxiety starting at 2.5y Increased α-syn levels in the SN, striatum, and cortex	[62]
	Rhesus monkeys (*Macaca fascicularis*)	CRISPR-Cas9 *PINK1* in oocytes	Decrease in grey matter density in the cortex Neurodegeneration in the SN, striatum, and cortex Increased astrogliosis	[63]
NA: non-attributable; y: year(s)				

## Data Availability

Not applicable.
